# Reductive quenching of photosensitizer [Ru(bpy)_3_]^2+^ reveals the inhomogeneous distribution of sites in PAN polymer nanofibers for light-driven redox catalysis†

**DOI:** 10.1039/d4ra05672h

**Published:** 2024-10-15

**Authors:** Svea M. Stepping, Nikita Vashistha, Sana Ullah, Poting Liu, Montaha Anjass, Benjamin Dietzek-Ivanšić

**Affiliations:** a Institute of Physical Chemistry, Friedrich-Schiller-University Jena Helmholtzweg 4 07743 Jena Germany benjamin.dietzek@uni-jena.de; b Department of Chemistry-Ångström, Physical Chemistry, Uppsala University 75120 Uppsala Sweden; c Leibniz Institute of Photonic Technology Jena, Department of Functional Interfaces Albert Einstein Allee 9 07745 Jena Germany; d Institute of Inorganic Chemistry I, Ulm University Albert-Einstein-Allee 11 89081 Ulm Germany; e Department of Chemistry, University of Sharjah 27272 Sharjah United Arab Emirates

## Abstract

Integration of molecular photocatalysts into redox-inert polymers constitutes a path towards photocatalytically active, lightweight materials. In particular, electrospun polymer fibers hold potential due to their favorable surface-to-volume ratio and their straightforward fabrication. This study focuses on the polyacrylonitrile (PAN) fibers, into which the prototype photosensitizer (PS) ruthenium tris(bipyridine) [Ru(bpy)_3_]^2+^, has been embedded by electrospinning. Studying the interaction between the optically excited [Ru(bpy)_3_]^2+^ with a non-redox inert solvent within the nanofibers, we resolve a distribution of microenvironments, which differ by the extent to which the photosensitizer is exposed to the solvent. This results in a non-exponential decay of the complex's emission and pronounced differences in the transient absorption signals.

## Abstract 2

The integration of molecular photocatalysts into redox-inert polymers constitutes a path towards photocatalytically active, lightweight materials while providing a distribution of microenvironments.

## Graphical abstract

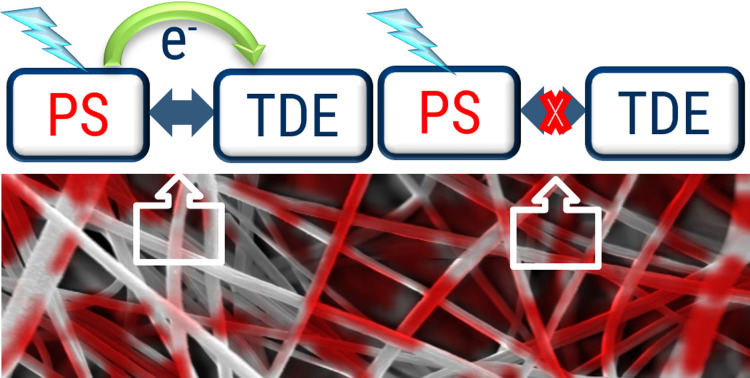

## Introduction

Heterogeneous catalysis, a critical discipline within chemical processes, entails catalysts residing in a separate phase from the reactants, typically as solids, which aids in effective separation.^1,2^ This form of catalysis plays a central role in a wide area of chemical reactions, especially those aimed at addressing pressing environmental issues, and therefore development of an efficient catalytic system is crucial.^3–5^ In this regard, nanofiber mats fabricated by electrospinning are a promising candidate as they provide nanometer dimension fibers with high surface area, reusability of catalysts, a larger number of active sites, and better performance than their powder counterparts.^6–8^ Furthermore, among the various methods for producing nanofibers, electrospinning is the most feasible and versatile, as it allows for rapid fabrication and scalability.^9^

Electrospun polymeric nanofibers have increasingly been used in photodegradation of organic pollutants, filtration, and generation of hydrogen in which they either act as a medium to immobilize the photocatalyst or a template or a sacrificial agent for photocatalysis.^10–12^ Thickness, porosity, adsorption, electrical conductivity, flexibility, and thermal stability are some of the parameters which can affect the performance of a nanofiber system.^12,13^ Chang *et al.* have demonstrated that a hollow TiO_2_ nanofiber provides a larger number of active sites (and thus shows better photocatalytic activity) than a solid nanofiber of the same material.^14^ So, active sites in a solid nanofiber are mostly situated on the outer interface and the photosensitiser embedded inside a solid nanofiber might not be accessible to the sacrificial donor/acceptor and this will govern the reduction and oxidation capabilities of the heterogeneous system. While electrospun nanofibers are now well explored, researchers have yet to study the effect of solvent accessibility on catalytic performance of the nanofiber.

For this study, we have immobilized ruthenium tris(bipyridine) complex in polyacrylonitrile (PAN) nanofibers fabricated by electrospinning to investigate the impact of immobilization on the excited state population. Ruthenium tris(bipyridine) [Ru(bpy)_3_]^2+^ is favored in light-driven catalysis due to its excellent photophysical properties, including a long-lived excited state and high absorption in the visible range. The history and significant impact of the metal-to-ligand charge transfer (MLCT) excited states in [Ru(bpy)_3_]^2+^ is noteworthy in the realms of photochemistry and photophysics.^15–17^ Damrauer *et al.* conducted the pioneering investigation of the excited state of [Ru(bpy)_3_]^2+^ in solution on a femtosecond scale suggesting that the metal-to-ligand charge transfer state (^3^MLCT) was established within 300 fs.^18^ According to Kallioinen *et al.*, sample preparation and experimental conditions can significantly affect this excited state population.^19^

In this work, we study steady-state absorption and emission and transient absorption behavior of ruthenium tris(bipyridine) [Ru(bpy)_3_]^2+^ in PAN nanofibers in the presence of a non-redox inert solvent. We specifically employ 2,2′-thiodiethanol (TDE) as a non-redox inert solvent, as it not only serves as a sacrificial electron donor to photoexcited [Ru(bpy)_3_]^2+^ but also matches the index of refraction of PAN. Thereby, it reduces optical scattering from the fibers, hence, facilitating the optical spectroscopic experiments.^20^ This study also demonstrates that electrospinning creates a heterogeneous distribution of [Ru(bpy)_3_]^2+^ and distinct microenvironments within nanofibers, limiting the accessibility of electron-donating solvents to the photosensitizer. These findings will be valuable while designing a photocatalytically active solid state nanofiber matrix.

## Results and discussion

**Fig. 1:**
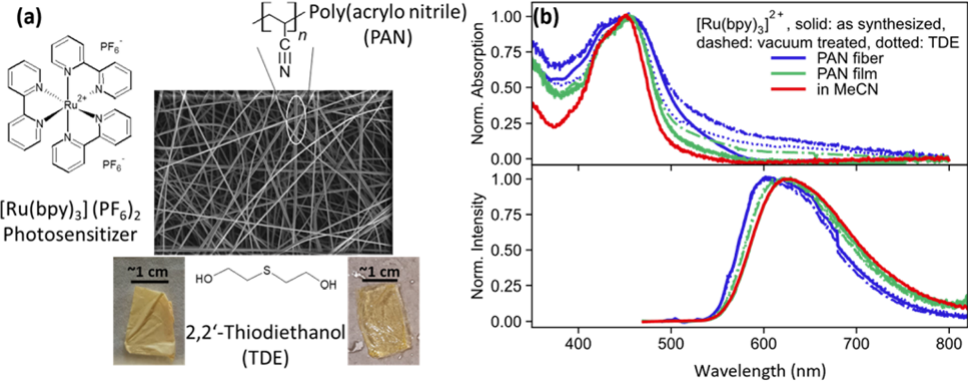
(a) SEM image of nanofibers made by electrospinning polyacrylonitrile (PAN) polymer. [Ru(bpy)_3_]^2+^ is incorporated in the fibers while fabrication and it imparts orange colour to sample. The fibers are made transparent with refractive index matching solvent 2,2′-thiodiethanol (TDE). (b) Normalized absorption (top panel) and emission spectra (bottom panel) for [Ru(bpy)_3_]^2+^ in film (green) and fiber (blue) sample with (dotted) and without (solid) TDE, vacuum treated (dashed) film and fiber sample with and without TDE, and in MeCN (red) as solution. The absorption spectra show broadening while emission spectra show a blue shift of emission in fibers and films.

Fig. 1(a) shows the SEM image of PAN fibers containing [Ru(bpy)_3_]^2+^ photosensitizer. The fibers have diameters between 400 nm and 1.2 μm and are opaque. For spectroscopic measurement, TDE is used as a non-redox inert-index matching solvent. Fig. 1(b) shows the normalized absorption and emission spectra of [Ru(bpy)_3_]^2+^ as dissolved in MeCN, integrated into electrospun PAN fibers, and drop-casted PAN films, with and without TDE. Furthermore, the fibers were studied as synthesized and after vacuum treatment (with and without TDE). The latter procedure was applied to remove DMF from the fiber samples, which is a residual from the electrospinning procedure. The removal of DMF through vacuum treatment was further confirmed by Fourier transform infrared spectrometer-attenuated total reflection (FTIR-ATR) analysis on the film and fiber sample conducted at a vacuum pressure of 80 kPa for 1 h (see ESI Fig. S1†).

As depicted in Fig. 1(b) (top panel), the characteristic metal-to-ligand charge transfer (MLCT) absorption peak of [Ru(bpy)_3_]^2+^ at about 450 nm is visible not only in MeCN solution but also in the fiber and film samples. Considering the latter, the MLCT band appears broadened when the complex is embedded into a PAN film. The broadening increases further in the electrospun PAN fibers. We ascribe this spectral broadening to an increase in intermolecular interactions and aggregation of the complex in the polymer. While these two effects might be considered similar in PAN films and fibers, the broader MLCT absorption band of [Ru(bpy)_3_]^2+^ in PAN fibers (FWHM = 8400 cm^−1^) as compared to PAN films (FWHM = 4400 cm^−1^) is considered an indication of the increased inhomogeneity of the fiber samples.^21,22^

Fig. 1(b) (bottom panel) also shows normalized ^3^MLCT emission spectra recorded upon 450 nm excitation. While in solution the emission of the complex peaks at 620 nm, the emission blueshifts to 600 nm in fibers. The shape of the emission spectrum is essentially unaffected by both vacuum treatment (Fig. 1(b) dashed curves) of the fibers, *i.e.* removal of residual DMF in the polymers left after electrospinning, and addition of TDE (Fig. 1(b) dotted curves). The blue shift of the emission upon polymer integration of the complex is attributed to the absence of polar solvent molecules in the vicinity of the complexes, which solvate and, hence, stabilize the emissive MLCT state in solution.^21^

**Fig. 2:**
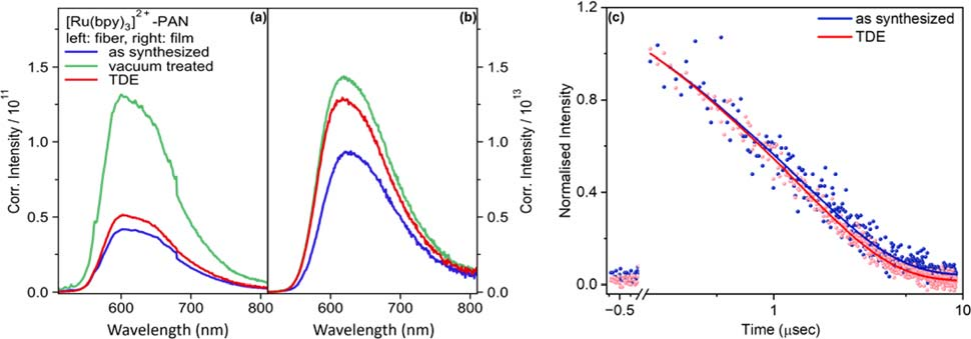
Corrected intensity emission spectra of (a) [Ru(bpy)_3_]^2+^ in PAN fibers and (b) in PAN film with (green) and without (blue) vacuum treatment and with (red) and without (blue) TDE. The samples under study contain the same concentration of [Ru(bpy)_3_]^2+^ and yield optical densities of *ca.* 0.35 and 0.08 at the MLCT absorption band for fiber and film samples, respectively. Emission and excitation measurements were performed under same conditions. (c) Normalized emission decay profile of fiber samples with and without TDE. In order to derive the kinetic traces, the emission has been spectrally integrated in the range between 520–800 nm.

While the spectral shape of the emission is not altered by vacuum treatment or addition of TDE, Fig. 2 reveals that the emission intensity for vacuum-treated fibers is approximately threefold higher than the emission intensity of the as synthesized fibers and after TDE addition. The lowest emission intensity is observed for fibers, which were characterized as synthesized. The reduction in emission intensity is more pronounced in fibers than films. This is because the porous nanofibers exhibit a higher accessibility of the quencher (TDE) to approach and interact with [Ru(bpy)_3_]^2+^. Nonetheless, the emission quantum yields for non-vacuum treated fiber samples irrespective of the addition of TDE vary between 22.0% – 20.8%, which is high compared to the complex in deoxygenated MeCN (9.5%).^23^

The emission quenching observed upon addition of TDE is ascribed to reduction of the photoexcited [Ru(bpy)_3_]^2+^.^24^ Using the tabulated values for the reduction potential of the excited state of [Ru(bpy)_3_]^2+^ ^25^ and the oxidation potential of TDE^26^ and Rehm–Weller equation (ESI†), we can state that the reductive quenching by TDE is thermodynamically favoured (=−2.07 to −2.69 eV). From quantum yield measurements, we approximate the fraction of molecules, which is shielded against reductive quenching by TDE to about 6%. This again points to the heterogeneity of the samples, *i.e.* not all the photosensitizers embedded into nanofiber can interacts with the quencher. Due to the uneven distribution, molecules will encounter distinct microenvironment, causing some photosensitizer molecules located deep within the polymeric nanofiber to be shielded from the electron-donating solvent.

The emission increase upon vacuum treatment of the fibers points to emission quenching by DMF, which is used to dissolve the PAN during the electrospinning, or dimethylamine (DMA), which is a decomposition product of DMF. Both DMF and DMA, with an oxidation potential of +1.900 V *vs.* SHE ≈ *vs.* NHE^27^ and +1.27 V *vs.* NHE,^28^ respectively, can also result in a favorable reaction free energy. The effect of residual DMF on the [Ru(bpy)_3_]^2+^ emission appears to be stronger in fibers compared to films. This is due to high porosity of the fibers (ESI Table 1†). Nonetheless, in the following, we will focus the discussion on the reductive quenching of the photosensitizers in the presence of TDE as a non-redox inert solvent.

Fig. 2(c) shows the bi-exponential decay of [Ru(bpy)_3_]^2+^ emission from as synthesized fibers in the presence and absence of TDE. The first lifetime measures approximately 0.2 μs, while the second lifetime is observed to be around 1.6 μs. Since there is negligible leaching of [Ru(bpy)_3_]^2+^ from the fibers under the current experimental conditions, we ascribe the bi-exponential decay to emissive decay of [Ru(bpy)_3_]^2+^ situated either on the surface or in the bulk of a nanofiber. So, immobilizing [Ru(bpy)_3_]^2+^ in PAN increases the lifetime compared to the complex being dissolved in MeCN (*ca.* 985 ns).^21^ Comparing the emission decay in the absence and presence of TDE, it becomes apparent that TDE accelerates the emission decay due to reductive quenching.

**Fig. 3:**
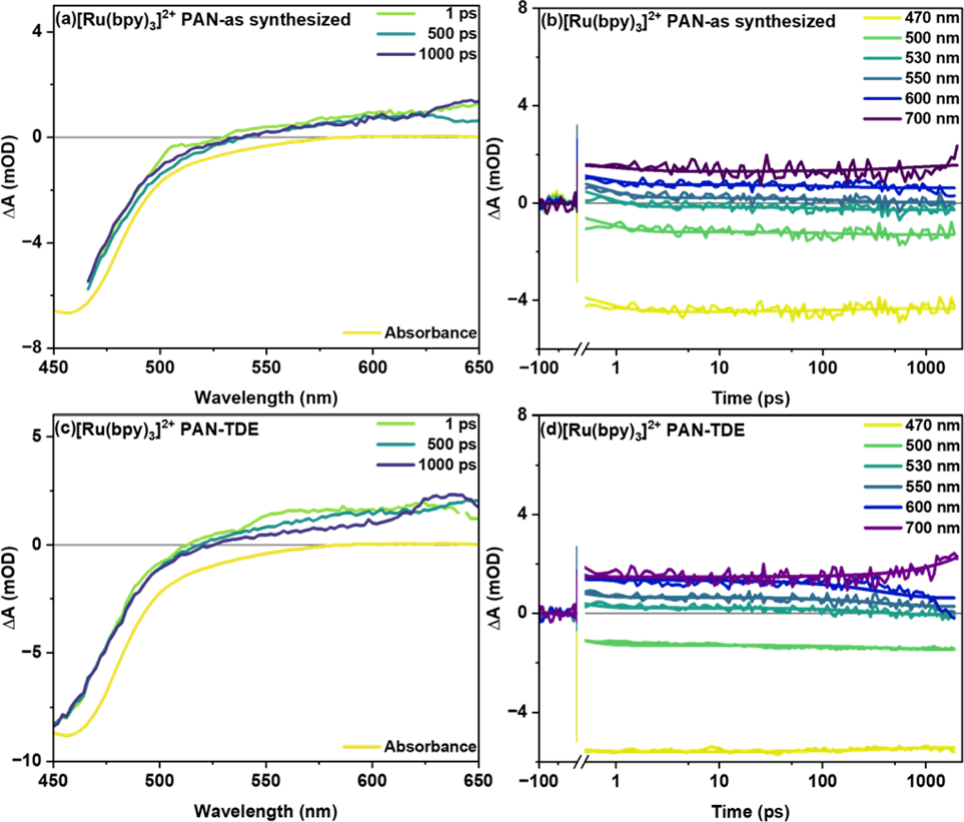
Femtosecond transient absorption spectra (with inverted steady-state absorption) at different delay time and kinetic traces at specific wavelengths of [Ru(bpy)_3_]^2+^ in PAN fiber samples (a and b) as synthesized and (c and d) with the non-redox inert solvent TDE. Both samples were excited with 400 nm pump (*P*_avg_ = 0.2 mW) and transient data was acquired with a white-light supercontinuum probe.

Fig. 3 shows transient absorption signals of the fiber samples without (as synthesized) and with TDE upon excitation of the MLCT transition of the photosensitizer at 400 nm. Both samples show a ground state bleach (GSB) centered at around 450 nm accompanied by a broad, spectrally unstructured excited state absorption (ESA) above 490 nm (Fig. 3(a) and (c)). This spectral shape is characteristic of MLCT states in Ru(ii) polypyridine complexes and arises from the superposition of the bleach of the ^1^MLCT transition and a ligand-to-metal transition (LMCT) originating from the formally reduced bipyridine ligand (bpy^.–^).^18^ In the as synthesized sample studied here, the temporal changes of the transient absorption spectra are minute (Fig. 3(b)). For sample with TDE (Fig. 3(d)), the transient absorption kinetics rather show a decrease of the ESA at longer delay times (starting at about 100 ps). This decrease in ESA, however, is not accompanied by a recovery of the ground state. The selective disappearance of the LMCT band indicates that the slow kinetic process visible in the data is associated with reductive quenching of the long-lived ^3^MLCT state. Consistent with these considerations is the fact that the slow decay of the ESA features is only observed for the fiber samples treated with TDE (the transient absorption data obtained for [Ru(bpy)_3_]^2+^ in MeCN does not show a decay of the spectral features either; see ESI Fig. S2†). Additionally, when comparing vacuum treated fibers with as synthesized fibers (see ESI Fig. S3†), a rapid reduction of ESA is observed after 1 ps (within 100 ps), which manifests itself as a widening of GSB feature at around 550 nm. This is likely caused by the reductive quenching of excited [Ru(bpy)_3_]^2+^ by residual DMF in the polymer.

In global fitting of decay kinetics for [Ru(bpy)_3_]^2+^ in PAN fibers, three distinct lifetimes are observed for as synthesized and with TDE samples. Firstly, a rapid decay (*τ*_1_ = 0.6 ps as synthesized and 0.3 ps with TDE), secondly, a somewhat slower decay component (*τ*_2_ = 160 ps as synthesized and 386 ps with TDE), thirdly, a non-decaying component (*τ*_3_ > 2 ns as synthesized and with TDE). The second decay *τ*_2_ is attributed to reductive quenching and/or solvent relaxation. The higher *τ*_2_ for with TDE is assigned to reductive quenching with TDE. The non-decaying component *τ*_3_ represents long lived charge carriers occupying the ^3^MLCT state. These will either radiatively or non-radiatively decay to ground state on a longer time scale.

## Experimental

### Sample preparation

Fiber samples were prepared by electrospinning of a *N*,*N*-dimethylformamide (DMF, Carl Roth) solution with dissolved polyacrylonitrile (PAN, Sigma-Aldrich, *M*_m_ = 150 000 g mol^−1^, 10 wt%) and [Ru(bpy)_3_](PF_6_)_2_ with bpy = 2,2′-bipyridine (Sigma-Aldrich, 0.25 wt%) onto aluminum foil (VWR International GmbH). In a horizontal electrospinning setup (Fluidnatek, LE-50), a voltage of 14 kV and a flow rate of 0.6 ml h^−1^ were applied to obtain a thickness of 700–800 nm. The resulting fiber-aluminum foil sheets were cut into pieces (*ca.* 1 cm^2^) and transferred to pre-cleaned (with acetone and ethanol) microscope glass slides (Menzel-Gläser). The aluminum foil was removed mechanically. Thin film samples were prepared by drop-casting a solution of DMF containing PAN and [Ru(bpy)_3_]^2+^ with the same weight ratio as in the fiber samples onto similar pre-cleaned glass slides without further treatment. 2,2′-Thiodiethanol (TDE, Sigma Aldrich, purity ≥ 95.0% (GC)) was added as a refractive index matching solvent to obtain transparent samples. Solutions of appropriate concentration of [Ru(bpy)_3_]^2+^ were prepared in acetonitrile (MeCN, Carl Roth), purged with argon (15 min), and measured in quartz cuvettes (*d* = 10 mm, Hellma). All chemicals were used without further purification.

### Steady-state absorption and emission spectroscopy

Steady-state absorption spectra of fiber samples were recorded on a JASCO spectrometer V-780, whereas the complex in solution was measured with a mono-beam spectrometer (SPECORD S 600, Analytik Jena). Absorption from the fiber samples, which did not contain non-redox inert solvent was collected through a BaSO_4_-coated integrating sphere (Ulbricht sphere) with a diameter of 150 mm and a built-in PMT detector. Steady-state emission spectra were measured with an Edinburgh instrument spectrophotometer (FLS980). A reflection configuration was used to measure emission in fiber and film samples while solution samples were recorded in an L-configuration. Emission spectra were energy corrected by multiplying the intensity detector readings with the wavelength squared.^29^ The absolute emission quantum yield of the fiber samples was determined through an integrating sphere.

### Femtosecond transient absorption spectroscopy (fs-TA)

Femtosecond transient absorption (fs-TA) spectra were recorded in the UV-vis range (350–700 nm) with a custom-built pump-probe setup as detailed in previous description.^30^ A titanium–sapphire (Ti:Sa) oscillator (Libra, Coherent Inc.) generates pulses at a fundamental wavelength of 800 nm with a repetition rate of 1 kHz. After frequency doubling by a BBO crystal a 400 nm pulse with 0.2 mW average power is used to excite the samples. Using a Berek compensator and polariser the relative polarisation of pump and probe beam was set to the magic angle of 54.7°. The pump scatter recorded at a delay time of −1 ps is subtracted from the data. Data processing was done through Python (v3.10 and v3.11) with the package KiMoPack.^31^ Before fitting, each data set was chirp-corrected to compensate for group velocity dispersion. Since TA data near time zero is prone to coherent artifacts^32^ we neglected the data around ±150 fs region in the data analysis.

## Conclusions

In this work, we have investigated the interaction of non-redox inert solvent with optically excited [Ru(bpy)_3_]^2+^ in redox-inert polymer polyacrylonitrile (PAN). Analysis of the emission data reveals that the emission from the [Ru(bpy)_3_]^2+^ in fiber samples with non-redox inert solvent TDE is only weakly quenched, *i.e.* not all excited [Ru(bpy)_3_]^2+^ are accessible to the solvent. This revelation highlights the distribution of complex in the fiber and heterogeneous nature of the sample. Quantum yield measurements indicate that the complex exhibits a consistently high quantum yield of 22.0% (as synthesized) and 20.8% (upon addition of TDE). A rough estimate based on the quantum yields shows that about 6% of excited [Ru(bpy)_3_]^2+^ molecules are accessible to quenching by TDE. Additionally, the emission lifetime shows a bi-exponential decay, with the dominant component lasting up to 1.6 μs. The reductive quenching by TDE becomes more apparent in fs-TA measurements, highlighting its impact at longer delay times (>100 ps). The results presented show that ensuring solvent accessibility of molecular components integrated into polyacrylonitrile presents a major challenge in designing photoredox catalytically active fibers.

## Data availability

Data for this article, including [csv, sia, tiff, txt, acsii, xlsx, dpt, prf, jwgbp, FS] are available at [FSU Jena cloud folder under TDE-Raw-Files] at [https://cloud.uni-jena.de/s/8stWzTWfXeYY37d].

## Conflicts of interest

There are no conflicts to declare.

## Supplementary Material

RA-014-D4RA05672H-s001
