# Supplementary material for: Reductive quenching of photosensitizer [Ru(bpy)3]2+ reveals the inhomogeneous distribution of sites in PAN polymer nanofibers for light-driven redox catalysis
Source: RSC Adv. 2024 Oct 15;14(44):32501–5. doi: 10.1039/d4ra05672h (PMC11474860; doi:10.1039/d4ra05672h)
Supplement: RA-014-D4RA05672H-s001 [file RA-014-D4RA05672H-s001.pdf]

Supplementary Information (SI) for RSC Advances.
This journal is © The Royal Society of Chemistry 2024

Supporting Information

Reductive quenching of photosensitizer $[Ru(bpy)_3]^{2+}$ reveals the inhomogeneous distribution of sites in PAN polymer nanofibers for light-driven redox catalysis

Svea M. Stepping[a,b], Nikita Vashistha[a,c], Sana Ullah[d], Poting Liu[a,c], Montaha Anjass[d,e], Benjamin Dietzek-Ivanšić[a,c*]

[a] Institute of Physical Chemistry, Friedrich-Schiller-University Jena, Helmholtzweg 4, 07743 Jena, Germany

Email: benjamin.dietzek@uni-jena.de

[b] Department of Chemistry-Ångström, Physical Chemistry, Uppsala University, 75120 Uppsala, Sweden

[c] Leibniz Institute of Photonic Technology Jena, Department of Functional Interfaces, Albert Einstein Allee 9, 07745 Jena, Germany

[d] Institute of Inorganic Chemistry I, Ulm University, Albert-Einstein-Allee 11, 89081 Ulm, Germany

[e] Department of Chemistry, University of Sharjah, 27272 Sharjah, United Arab Emirates

Table 1: Average (Avg.) pore diameter and surface area of PAN Fibers samples measured through

	PAN fiber	$[Ru(bpy)_3]^{2+}$ PAN fiber as synthesized	$[Ru(bpy)_3]^{2+}$ PAN fiber TDE	$[Ru(bpy)_3]^{2+}$ PAN fiber TDE vacuum treated
Avg. pore diameter /nm	25.8	11.7	15.4	24.8
Surface area / (m^2/g)	232.8	644.3	505.4	325.7

BET (samples are pre-degassing at 100 ℃ for 120 min).

The specific surface area and average pore diameter of PAN fibers were estimated using the Brunauer–Emmett–Teller (3P instrument, Micro 200) method with N_2 as absorbate. From the results (Table 1) we can see that the plain PAN fibers have a large pore diameter of 25.8 nm and a surface area of 232.8 m^2/g. With the addition of $[Ru(bpy)_3]^{2+}$ pore diameter shrunk to 11.7 nm, however, the surface area tripled, indicating that the synthesized fibers can absorb three times the amount of N_2 compared to plain PAN fibers. The addition of the non-redox inert solvent (TDE) causes the fibers to swell and fill the pores, reducing the available surface area by 138.9 m^2/g and decreasing the average pore diameter[1]. On vacuum treatment of these fibers, the excess DMF is removed from the fibers which results in the enlargement of pore size which reaches a value similar to plain PAN fiber. Due to the removal of DMF, TDE will percolate more, reducing the available surface area to 325.7 m^2/g.

Therefore, the vacuum-treated sample with TDE will have significantly less surface area for adsorption compared to the non-vacuum-treated sample.

To assess whether TDE can reduce the excited state of $[Ru(bpy)_3]^{2+}$, the reaction free energy can be calculated using the Rehm-Weller equation[2,3]:

$$\Delta G = \Delta E\left(Ru(II)^*/Ru(I)^*\right) + \Delta E\left(TDE/TDE^+\right) - \Delta E_{00} + \nu - \Delta E_{00} = [+1.01 + (-1.03\ to - 1.63)] - 2.07 =- 2.07 \quad (1)$$

Where ΔE_{00} is energy difference between ground and excited state in eV which is calculated at 600 nm absorption tail. The values of reduction potential for excited state of $[Ru(bpy)_3]^{2+}$ is 1.01 V vs. NHE[4] and TDE is 1.03 V–1.63 V vs. NHE[5]. The term w_1 depends on electron transfer distance between donor and acceptor and can be considered as '0' is electrostatic

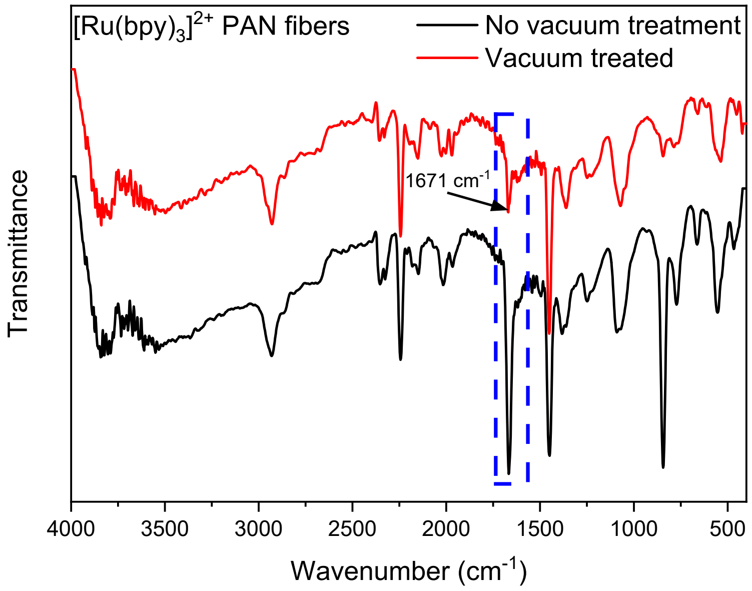

complex occur before charge trasnfer[4].

Figure S1: Fourier transform infrared spectrometer - attenuated total reflection (FTIR-ATR) spectra of a non-vacuum and vacuum treated [Ru(bpy)$_3$]$^{2+}$ PAN fiber samples exposed to -80 kPa pressure for 1 hour.

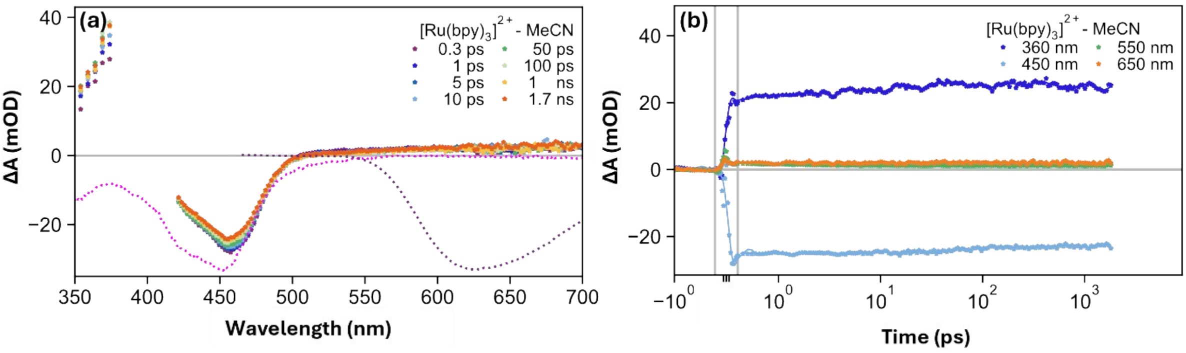

FTIR-ATR (Bruker, ALPHA-II, Platinum-ATR) was used to analyze the existence of DMF in electrospun fibers. The ATR spectra show that the peak at 1671 cm^{-1}, which is characteristic C=O bond strength of DMF solvent[6], is present in both samples (before and after vacuum treatment) without any shift. However, a significant shrinkage in transmittance was observed in the vacuum-treated sample. It is strengthening our assumption of the presence of DMF solvent in the electrospun fibers. However, applying high pressure (Vacuum treatment) helps in removing the extra solvent from the fibers.

Figure S2: Photophysical properties of $[Ru(bpy)_3]^{2+}$ in argon purged (15 min) MeCN. a) Transient absorption spectra at selected delay times. The sample was excited by 400 nm pump (P_{avg}=0.2 mW) and the absorption behavior was observed with a WL probe. The inverted dotted curve represents state steady-state absorbance (magenta) and emission (purple), respectively. b) transient kinetics at key wavelengths. The kinetics were recorded up to a delay time of 2000 ps.

Figure S2 shows the transient absorption behavior of the $[Ru(bpy)_3]^{2+}$ in MeCN. The negative signal centered at 450 nm corresponds to MLCT and is consistent with the steady-state absorption curve (dotted curve (magenta)). The ESA behavior below 400 nm and above 500 nm is either due to absorption from reduced bipyridine ligand or LMCT transition[7]. Transient behavior shows a prominently non-decaying ESA within the measurement time window.

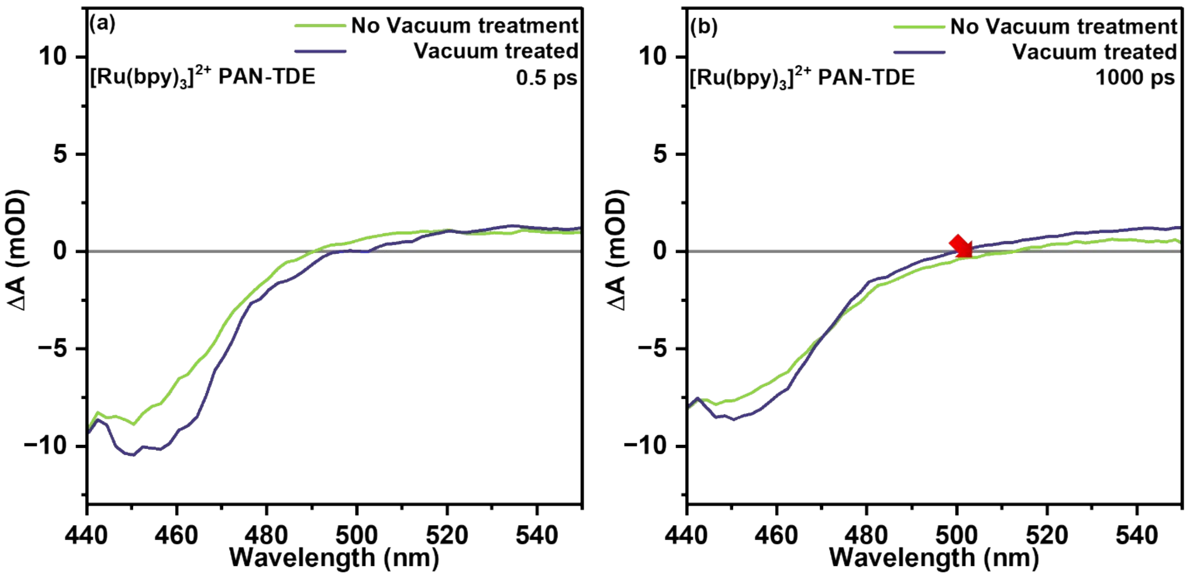

Figure S3: Transient absorption spectra of as synthesized and vacuum-treated $[Ru(bpy)_3]^{2+}$ PAN-TDE fiber sample a) at 0.5 ps and b) 1000 ps delay time. The sample was exposed to -60kPa pressure for 1 hour and then we added TDE to it. For transient absorption study the sample was excited by 400 nm pump having 0.2 mW average power.

Figure S3 compares the difference in absorption(ΔA) spectra of as synthesized and vacuum treated sample at fixed delay times (0.5 ps and 1000 ps). After 1000 ps, the as synthesized sample that was not subjected to vacuum treatment exhibited a decay of ESA (or a consequent widening of GSB) around 500 nm. This decay was not observed in the vacuum-treated fiber. The reason is that vacuum treatment removes the DMF from the fibers, which is responsible for reducing the excited state of $[Ru(bpy)_3]^{2+}$.

References

1. S. Chatterjee and S. De, *Environmental Science: Water Research & Technology*, 2015, **1**, 227–243.
2. D. Rehm and A. Weller, *Israel Journal of Chemistry*, 1970, **8**, 259–271.
3. A. A. Taherpour, *Arabian Journal of Chemistry*, 2017, **10**, S609–S616.
4. C. K. Prier, D. A. Rankic and D. W. C. MacMillan, *Chemical Reviews*, 2013, **113**, 5322–5363.

5. H. Mohan and J. P. Mittal, *International Journal of Radiation Applications and Instrumentation Part C Radiation Physics and Chemistry*, 1991, **38**, 45–50.
6. Y. K. Ren, S. D. Liu, B. Duan, Y. F. Xu, Z. Q. Li, Y. Huang, L. H. Hu, J. Zhu and S. Y. Dai, *Journal of Alloys and Compounds*, 2017, **705**, 205–210.
7. N. H. Damrauer, G. Cerullo, A. Yeh, T. R. Boussie, C. V. Shank and J. K. McCusker, *Science*, 1997, **275**, 54–57.
